# Food-Grade Microwave-Assisted Depolymerization of Grape Seed Condensed Tannins: Optimizing the Reaction Using Gallic Acid as a Nucleophile

**DOI:** 10.3390/polym17050682

**Published:** 2025-03-04

**Authors:** Carolina F. Morales, Fernando A. Osorio

**Affiliations:** Department of Food Science and Technology, Technological Faculty, University of Santiago, Chile (USACH), Av. El Belloto 3735, Estación Central, Santiago 9170022, Chile; carolina.moralesc@usach.cl

**Keywords:** condensed tannins, depolymerization, antioxidants, SN1 reaction

## Abstract

Food waste has a significant social impact but can be revalued as a source of bioactive compounds, such as condensed tannins. This abundant biomass, corresponding to a polymeric antioxidant, must be depolymerized to become bioavailable. Previous studies have investigated polymer degradation into oligomers using high temperatures and expensive nucleophiles, often under conditions unsuitable for food applications. In the present investigation, it is proposed that the depolymerization of condensed tannins can occur under food-grade conditions using a Generally Recognized as Safe (GRAS) solvent by optimizing the reaction’s heating method with microwave assistance and using gallic acid as a nucleophile. Thermal studies indicate that the degradation of total polyphenols content follows first-order kinetics and occurs above 80 °C in microwave. Depolymerization follows second-order kinetics, yielding epicatechin as the primary product with zero-order formation kinetics. The optimized factors were 80% *v*/*v* ethanol, 10 mg/mL polymeric tannins, and 5.88 mg/mL gallic acid. Under these conditions, the reaction efficiency was 99.9%, the mean particle diameter was 5.7 nm, the total polyphenols content was 297.3 ± 15.9 EAG mg/g, and the inhibition of ABTS●+ and DPPH● radicals was 93.5 ± 0.9% and 88.2 ± 1.5%, respectively. These results are promising for future scaling processes.

## 1. Introduction

Agri-food industries generate large quantities of processing waste, such as peels, seeds, leaves, and stems, among the most common waste generated in processing fruits and vegetables [1]. Landfilling, composting, and incineration are the popular options for most of this waste [2]. Around 50% of these losses come from fruits and vegetables [3]. However, this type of bioproduct has been undervalued since it is notably rich in polyphenolic compounds, the most important class of natural antioxidants [4] with biological activities promoting health and preventing diseases [5].

Therefore, valorizing waste and food losses is not just an issue of high social impact, but also a significant step towards sustainable waste management. It has become a promising technological and ecological solution, especially for developing products with high added value, such as nutraceuticals or food supplements that benefit human health [6].

According to official figures from the World Health Organization (WHO), cancer is the leading cause of death in the world. In 2020 alone, almost 10 million deaths were attributed to this disease, and around a third of these were attributed to a low intake of fruits and vegetables [7]. The above, added to the current lifestyle, which is characterized by stress and the intake of harmful substances, causes a significant increase in long-term semi-pathological conditions such as oxidative stress, one of the leading causes of chronic diseases related to cancer. This underscores the urgent need for research and innovation in the field of health-promoting ingredients [8].

Proanthocyanidins or condensed tannins (CT) are natural polymers with molecular weights of up to 30,000 Da whose structural unit is flavan-3-ol, mainly catechin (Ca) and epicatechin (Ep), linked by interflavanic bonds [9]. These metabolites are widely available in the plant kingdom; it is estimated that they are the fourth most abundant type of biomass material [10], and grape seeds contain one of the richest sources of CT [11], grapes (*Vitis vinifera* L.) being one of the most abundant fruit crops in the world, with an annual production of 75.8 million metric tons [3].

Numerous studies have demonstrated the broad spectrum of bioactivity of CT, mainly their antioxidant, anti-inflammatory, anti-neurodegenerative, antimicrobial, anticancer, and cardioprotective activity [12,13,14,15,16]. However, its biological activities are related to its main degree of polymerization (mDP). It has been reported that monomeric and oligomeric units of up to 5 flavan-3-ol units can pass into the bloodstream. In comparison, polymeric units (about five monomers units) are not absorbable through the gastrointestinal tract [17], because of their absorption represents around 1% in humans [13,18]. Therefore, it is an emerging challenge to develop effective methods to convert condensed tannins into bio accessible small molecules [19].

The depolymerization of CT is carried out through a unimolecular nucleophilic substitution reaction (SN1), where the first step and determining the reaction rate is the formation of the carbocation upon breaking the interflavanic bond, which is activated by increasing the temperature above 65 °C in the presence of H^+^. The second step consists of the reaction between the carbocation formed and the nucleophile (Nü), generally a thiol or phloroglucinol, which must be in excess [20,21,22].

Recently, the depolymerization of CT to oligomers has been studied to increase their bio accessibility, using procedures with high temperatures and the use of nucleophiles of high commercial value such as catechin (Ca), epicatechin (Ep), epicatechin gallate (EpG) [23,24,25], captopril [19], tiopronin [26], and sulfite/catechin mixtures [27]. In addition, these reactions have been carried out in conditions not suitable for food purposes since they use methanol as a solvent, leading to the formation of adducts that are not found in nature.

Due to its prevalence in the plant kingdom, gallic acid (GA) is a highly accessible and low-cost phenolic acid; therefore, it has been widely used as a standard to determine the total polyphenol content (TPC) of various analytes, taking advantage of the Folin–Ciocalteau assay [4]. In this context, GA has been widely recognized as an effective antioxidant, with the potential to be used in the food industry to reduce lipid oxidation [28] and manufacture bioactive antioxidant packaging [29,30], among others.

In this study, we propose a method for depolymerizing polymeric condensed tannins (P) that can be implemented in a food-grade setting using a Generally Recognized as Safe (GRAS) solvent. By optimizing the heating process of the reaction with eco-friendly technologies like microwave assistance (M) and the use of an accessible and low-cost Nü, we aim to produce natural monomers with high antioxidant capacity. These monomers could be a significant addition to the food industry’s arsenal of health-promoting ingredients, with the potential to prevent chronic diseases related to cancer.

## 2. Materials and Methods

### 2.1. Chemicals and Reagents

Grape seed CT was obtained from Laffort (Bordeaux, France). GA for synthesis was obtained from Merck (Darmstadt, Germany). Sodium hydroxide reagent grade ≥98% (NaOH) pellets (anhydrous), sodium acetate, acetic acid, Cysteamine hydrochloride (Cys), 2,2′-azino-bis-3-ethylbenzothiazoline-6-sulfonic acid diammonium salt (ABTS●+), 2,2-diphenyl-1-picrylhydrazyl (DPPH●), analytical standard of (+)-catechin and (−)-epicatechin, and primary reference standard of epicatechin gallate and catechin gallate (purity ≥ 99%) were obtained from Sigma-Aldrich (Sigma-Aldrich, Steinheim, Germany). Ethanol pa (purity ≥ 99.9%) from Merck KGaA (Darmstadt, Germany), hydrochloric acid from Merck (Darmstadt, Germany), Milli-Q water (Elga Purelab^®^ system, Griefswald, UK), and deionized water were used as solvents.

### 2.2. Characterization Extract of Condensed Grape Seed Tannins and Their Reaction Products

#### 2.2.1. Total Polyphenol Content (TPC)

TPC was determined by the Folin–Ciocalteu method using the procedure described by Wang et al. (2016) [16], in which the Folin–Ciocalteu reagent must be used and then measure the absorbance at 760 nm in a spectrophotometer. The result of this measurement is expressed as the content of total phenols equivalent to gallic acid per gram of dry-weight polyphenol (GAEmg/g) and its equivalent molar concentration of gallic acid (GAEmol/L).

#### 2.2.2. Structural Monomers Analysis and Mean Degree of Polymerization (mDP)

In this study, we aimed to analyze the depolymerization of CT. To achieve this, we followed the standardized protocol of Bianchi et al. (2016) [21] with slight modifications. This protocol involves the use of thiolysis reactions using Cys and hydrochloric acid (HCl). Specifically, 0.5 mg/mL of P was dissolved in methanolic hydrochloric acid pH 0.5 containing 25 mg/mL of Cys and placed in a thermostatic bath (TB) under a hood for 1 h at 60 °C. The reaction was stopped immediately by placing the reaction vessel in a water/ice bath and adding NaOH 5N until pH 3 was reached. The products were liofilized and then stored at −20 °C for later analysis.

The determination of the monomers structural units and the mDP of the CT was carried out with precision and reliability using a UHPLC/UV (UHPLC; Thermo Scientific Dionex UltiMate 3000, Waltham, MA, USA) analysis. This method employs a wavelength of 280 nm to determine the molar concentration (mol/L) of each flavan-3-ol monomer released from the condensed tannins. The mDP of CT could be estimated using Equation (1) [21].

(1) $$mDP=\frac{\sum \left[Fext\right]+\sum \left[Fter\right]}{\sum \left[Fter\right]}$$

where ∑[Fext]: molar concentration (µmol/L) of the total flavan-3-ol extension units released (flavan-3-ol/thioethers) after thiolysis; ∑[Fter]: molar concentration (µmol/L) of the total terminal flavan-3-ol units released (native flavan-3-ol) after depolymerization.

#### 2.2.3. Chromatographic Analysis by Ultra-High-Performance Liquid Chromatography (UHPLC)

Samples were analyzed by reverse-phase high-performance liquid chromatography (UHPLC; Thermo Scientific Dionex UltiMate 3000, Waltham, MA, USA). Samples were filtered through a 0.22 μm PTFE filter and separated on a C18 column (5 μm, 4.6 × 150 mm, Thermo Scientific, Waltham, MA, USA). The system contains a quaternary pump, an autosampler, a column thermostat, an ultraviolet (UV) detector, and a photodiode array detector (DAD).

For the quantification of the condensed tannins and reaction products, the chromatographic conditions were an injection volume of 10 μL, a flow rate of 1 mL/min, and a column temperature of 30 °C. The mobile phase consisted of phase A, trifluoroacetic acid/water (0.1:100 v:v), and phase B, water/acetonitrile (1:4 *v*/*v*) containing 0.08% trifluoroacetic acid. The detection of polyphenols obtained was carried out at 280 and 545 nm. The peaks were integrated into the chromatograms, and the external standard method was used for quantification of the concentrations of the reaction products.

#### 2.2.4. Structural Free Radical Scavenging Activity

ABTS●+ (2,2′-azino-di-[3-ethylbenzothiazoline sulfonate]) radical scavenging capacity was performed using absorbance measurements [31], which is expressed as a percentage of inhibition of ABTS Equation (2).

(2) $$\%ABTS\;inhibition=\frac{A_{control}-A_{mixture}}{A_{control}}*100$$

The DPPH● assay was performed according to the procedure described by Toro-Uribe et al. (2019) [18], which is expressed as a percentage of DPPH inhibition (Equation (3)).

(3) $$\%DPPH\;inhibicion=\frac{A_{control}-A_{mixture}}{A_{control}}*100$$

where $A_{control}$: absorbance of the mixture of ethanol and ABTS●+ solution at 734 nm or DPPH● at 517 nm; $A_{mixture}$: absorbance of the sample solution mixture.

#### 2.2.5. Mean Particle Diameters (MPD) and Polydispersity Index (PDI)

The MPD and PDI were measured using a dynamic light scattering instrument (DLS) (Litesizer 500, Anton Paar, Ashland, VA, USA) [32]. Before analysis, the suspensions were diluted with an appropriate buffer (sodium acetate and acetic acid buffer; 0.1 M; pH 3.0 ± 0.1) to prevent multiple dispersion effects.

### 2.3. Studies of Grape Seed Condensed Tannins’ Thermal Resistance and Microwave Behavior (CT)

The heating effect on CT was studied by comparing a thermostatic bath (TB) and a Microwave Reaction Platform (M) (MULTIWAVE 5000 Platform with 20SVT Rotor capacity 20 PTFE vessel, Anton Paar, Ashland, VA, USA). For this analysis, a 3^2^ × 2^1^ multilevel factorial experimental design was used (Table 1). A total of 1 mg/mL of CT was dissolved in 50% ethanol pH 3 and heated for 60 min at 40, 60, and 80 °C in both instruments, i.e., TB and M, respectively, taking samples every 5 min. The effect of temperature on the suspension’s TPC was analyzed according to the previously described methodologies.

### 2.4. Chemical Depolymerization of Grape Seed Tannins by Depolymerization with Gallic Acid

#### 2.4.1. Food-Grade Depolymerization of Grape Seeds Condensed Tannins (CT)

Depolymerization was carried out through unimolecular nucleophilic substitution (SN1) reactions [20,21] using gallic acid as a chain-disrupting nucleophile with food-grade modifications. According to the experimental design described below, different masses of condensed tannins were dissolved in aqueous ethanol with HCl pH 0.5 and dispersed by sonication for two minutes. Some reaction parameters were set according to the results previously obtained in this study and by other authors to analyze the effect of the solvent change; for this, the temperature was 60 °C, and the pH was 0.5 [21,24,33].

After the addition of different masses of gallic acid and a 60 min incubation in a Polytetrafluoroethylene (PTFE) vessel for microwave synthesis, the reaction was brought to a halt. This was achieved by immediately placing the reaction vessel in a water/ice bath and carefully adjusting the pH to 3 by adding 5 N NaOH. The final suspensions of depolymerized condensed grape seed tannins (P-Dep) were then lyophilized and stored at −20 °C for further analysis.

#### 2.4.2. Optimization of the Chemical Depolymerization Reaction

The depolymerization optimization procedure was carried out by studying the effect of the solvent and its interaction with the reagents (solvation capacity) using aqueous ethanol in different volume/volume percentage %(*v*/*v*) (S), CT, polymers from now on (P) and GA with variable concentrations. A 5^1^ × 2^2^ multilevel factorial experimental design was proposed, as described in Table 2. Once the reaction is optimized, its kinetics will be studied by reproducing the optimal depolymerization methodology and taking samples at different times up to 60 min of the reaction.

The response variable Reaction Yield (RY) is calculated according to Equation (4), considering the results obtained by UHPLC detailed in Section 2.2.3.

(4) $$RY=\frac{\left[P_{A}\;]-[P_{R}\right]}{\left[P_{A}\right]}*100$$

where RY: percentage of reaction yield (%); $\left[P_{A}\right]$: molar concentration of added polymers (mol/L); and $\left[P_{R}\right]$: molar concentration of remaining polymers (mol/L).

### 2.5. Optimized Depolymerization Analysis

#### 2.5.1. Kinetics Reaction Analysis

The kinetics of the reaction were analyzed by plotting the change from polymeric to monomeric flavan-3-ol. Therefore, the depolymerization kinetics were studied based on P_A_, P_R_, GA, and Ep as a molar concentration as a function of time were followed. Typical mathematical models for reaction kinetics were used. The fit of the models was verified using the coefficient of determination (R^2^) using CurveExpert Professional software (version 2.7.3., Hyams, D. G.), where $P_{A}$: molar concentration of added polymers (mol/L); P: polymeric condensed tannins (mol/L) $P_{R}$: number of polymers remaining after depolymerization (mol/L); and P−Dep: amount of all species in suspension after depolymerization (mol/L).

#### 2.5.2. Kinetics Color Measurement

Color analysis of condensed tannins and reaction products at different reaction times was performed using a HunterLab colorimeter (HunterLab, Reston, VA, USA), which uses a D65 illuminant to simulate daylight to achieve a homogeneous measurement; the samples were placed in a thin layer of 3 mm on a white background.

Color was expressed in terms of the parameters L*, a*, and b* according to the CIELab scale. The color difference (∆E*) between the P and P-Dep suspensions control was calculated using Equation (5).

(5) $$\Delta E^{*}=\sqrt{{\left(\Delta L^{*}\right)}^{2}+{\left(\Delta a^{*}\right)}^{2}+{\left(\Delta b^{*}\right)}^{2}}$$

where (∆E*): total color difference between the P suspension sample and the P-Dep suspension reaction product; (∆L*): luminosity difference between the P suspension sample and the P-Dep suspension reaction product; (∆a*): change in green–red color between the P suspension sample and the P-Dep suspension reaction product; and (∆b*): change in blue–yellow color between the P suspension sample and the P-Dep suspension reaction product.

The color intensity of the samples was calculated through the difference in the chroma (∆C*) values using Equation (6).

(6) $$\Delta C^{*}=\sqrt{{\left(\Delta a^{*}\right)}^{2}+{\left(\Delta b^{*}\right)}^{2}}$$

### 2.6. Statistical Analysis

All statistical analyses were performed using STATGRAPHICS Centurion (XVI v.16.1.03 software, StatPoint Technologies, Inc., Warrenton, Ashland, VA, USA). The results were expressed as mean values ± standard error. Differences were tested with ANOVA. The confidence level was set at 95% (*p* < 0.05). The model’s fit was verified using the coefficient of determination (R^2^), and the standard deviation and residual analysis program were used.

## 3. Results

### 3.1. Characterization Extract of Grape Seed Condensed Tannins

#### 3.1.1. Physicochemical Characterization

Characterization of P was carried out according to the methodologies previously described, which are summarized in Table 3.

#### 3.1.2. Determination of Molecular Structures and Mean Degree of Polymerization

**Figure 1:**
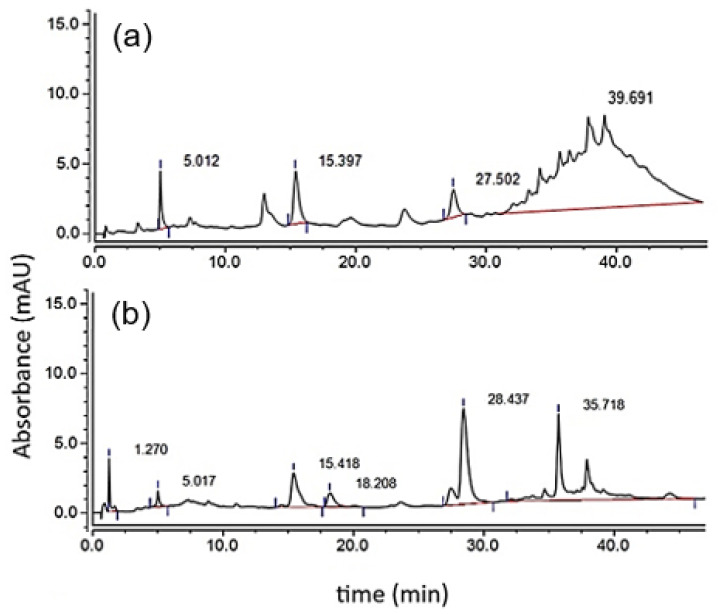
UHPLC/UV chromatograms at a wavelength of 280 nm of P (**a**) initial (P) and (**b**) after thiolysis depolymerization (P-Tiol).

The analysis of molecular structures and average degree of polymerization was carried out following the methodology described in Section 2.2.2 and Section 2.2.3, in which the chromatograms of Figure 1 and described in Table 4 were obtained.

Chemical structures obtained by thiolysis are shown in Table 4.

#### 3.1.3. Studies of the Effect of Temperature on the TPC in P Comparing 2 Heating Methods: Microwave (M) and Thermostatic Bath (TB)

The results of the experimental design proposed in Section 2.3 are shown in Table 5.

The corresponding statistical analysis are shown in Table 6.

#### 3.1.4. Evaluation of the Fit of the Kinetic Model of TPC Decay Under Treatment Conditions

**Figure 2:**
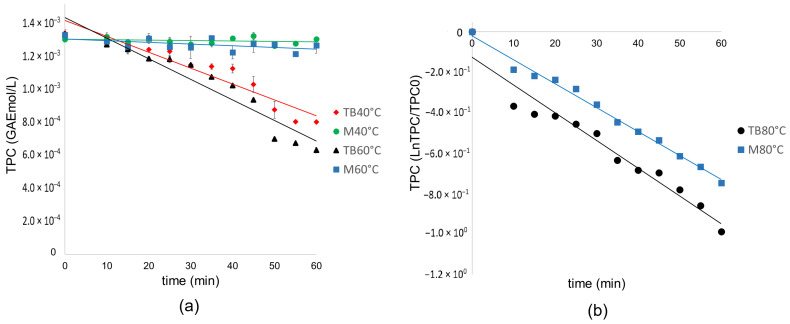
Linearized graphs for TPC on P as a function of time for (**a**) TB 40 °C, M40 °C, TB 60 °C, and M60 °C and (**b**) TB60 °C and M60 °C.

The TPC results were adjusted as a function of time for the different temperature levels and heating methods, as shown in Figure 2 and Table 7.

The adjustment of the results is shown in Table 7.

### 3.2. Chemical Depolymerization of P with GA

#### 3.2.1. Feasibility of Depolymerization of P Whit GA and Reaction Optimization

Two response variables and three experimental factors were specified as described in Table 2.

The selected design has 20 runs, with 1 sample to be taken in each run. The default model is cubic with 10 coefficients, as shown in Table 8.

Table 9 shows the ANOVA analysis for Reaction Yield (RY) and Mean Particle Diameter (MPD); this analysis considers the effect of the variables separately, as well as their interactions.

The statistical models were adjusted to the response variables. Two models with *p*-values below 0.05 indicate that the fitted model is statistically significant at 5.0%. Also, the R-squared statistics show the percentage of variation in the response that the adjusted model has explained. The R-squared values varied from 67.59% to 83.21%.

The optimal characteristics of the experimental factors were determined and established for each factor: S = 80% (*v*/*v*); P = 3.4 × 10^−2^ (mol/L) = 10 (mg/mL); and GA = 3.4 × 10^−2^ (mol/L) = 5.88 (mg/mL), establishing an optimal proportion P/S = 0.1 (% *m*/*v*) and P/GA 1.0 (mol/mol). With these characteristics, the response variables generate a desirability index of 100%.

#### 3.2.2. Optimized Depolymerization Kinetics Analysis

**Figure 3:**
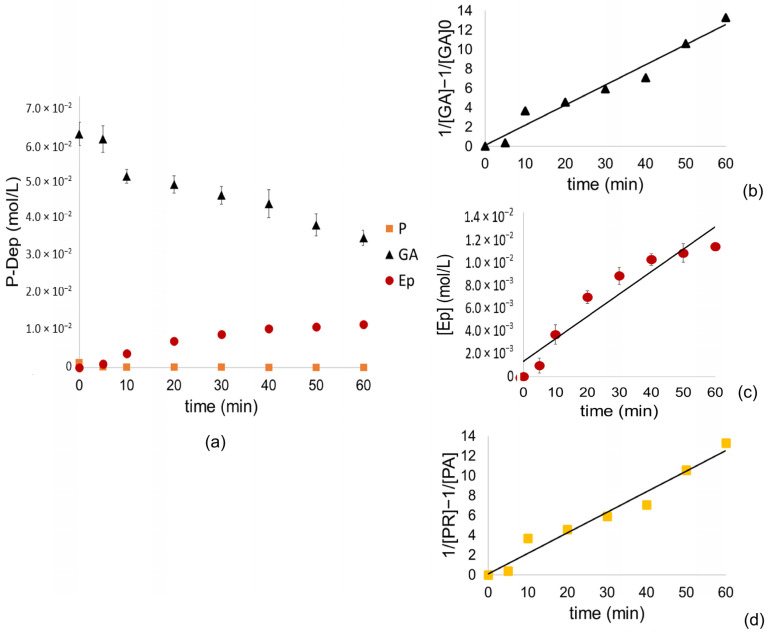
Molar concentration graphs as a function of depolymerization time for (**a**) GA, Ep, and P; fitted graphs with linearized models for (**b**) GA, (**c**) Ep, and (**d**) remaining polymers (P_R_). All the molecules studied are in a mixed depolymerization products suspension (P-Dep).

The concentrations of P (where added polymers PA and remaining polymers PR), GA (from the suspension of P), and the main depolymerization product of Ep were analyzed over time in the mix suspension of depolymerized P (P-Dep), along with their respective linearized models, as shown in Figure 3.

The equations corresponding to the linearization of the molecules studied in the reaction are presented below in Table 10.

#### 3.2.3. Optimized Depolymerization Color Analysis

**Figure 4:**
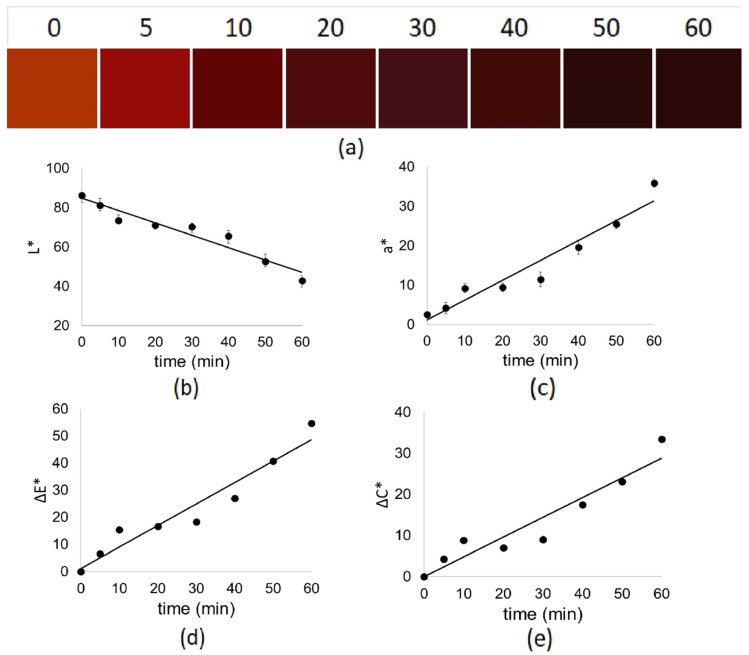
Color parameters change graphs as a function of depolymerization time for (**a**) visual color (photography of P-Dep the samples), (**b**) L*, (**c**) a*, (**d**) ∆E*, and (**e**) ∆C*.

Changes in color parameters were analyzed over reaction time for P-Dep, as shown in Figure 4.

Linear regressions for the color parameters during depolymerization are shown in Table 11.

### 3.3. Antioxidant Capacity

The antioxidant capacity of P, GA, and P-Dep was evaluated using the tests described in Section 2.2.4, which are shown in Table 12.

## 4. Discussion

### 4.1. Discussion of Characterization Extract of Grape Seed Condensed Tannins

The physicochemical characterization of P indicates that the TPC, approximately 230 GAEmg/g, is a high value since according to the portal antioxidants from the Institute of Nutrition and Food Technology (INTA), Chardonnay, Black seedless, and Crimson grape samples have a TPC of 200, 94, and 53 GAEmg/g in dry weight, respectively [34]. These results are representative of a high antioxidant activity close to 90% inhibition of ABTS●+ and DPPH● radicals, which is due to a high content of equivalent antioxidants of Trolox 8.8 ± 0.3, 9.2 ± 0.7, 9.4 ± 0.6 (mmol/L) for P, P-Dep, and AG, respectively, in agreement with Xu and collaborators, who analyzed the pomace of different grape varieties, finding equivalent antioxidant activities of Trolox of para varieties Viognier 3.5 and Vidal Blanc 7.7 µmol/g [35].

The MPS polymeric dispersions is in a relatively dispersed range with an IPD of 3.5, indicating the presence of P from monomers to at least octamers, consistent with 16.6 µm particle size, an average mDP of 7. 5.

Calibration curves were carried out with an external standard of the main components present in condensed grape seed tannins (P) as reported in the literature to identify the species corresponding to each peak in the chromatograms corresponding to both P and P-Dep: GA (5.0 min), Ca (15.4 min), and Ep (27.5 min) [36]. Additionally, Cys (1.3 min) and thiolated adducts of catechin (18.2 min) and epicatechin (28.4 min) were identified [21] (Table 4). These results are consistent with what was reported by Bianchi and collaborators in their Bioprotocol to determine the mDP of condensed tannins in which it is specified that, although there are no external standards for thiolated compounds, these appear immediately after the monomer with which they formed the adduct [21]. The final peak is seen with a retention time between approximately 35 and 45 min (Figure 1a); these multiple peaks correspond to the condensed tannins fractions—that is, to flavan-3-ol polymers [37,38]. Furthermore, the 28.4 min area increase can be seen (Figure 1b), which corresponds to the thiolated adducts of extensional Ep, confirming that Ep is the main monomer of the P under study. This peak overlapped with terminal Ep, so they had to deconvulse [39].

Table 4 identifies the percentage of added P ($P_{A}$) contained in the tannin samples, which corresponds to more than 90% of the total mass. The main degree of polymerization (mDP) of 8, which is indicative that this fraction of the sample is highly polymeric [13,40]. Furthermore, the depolymerization reaction yield with cysteamine under these conditions was 57.5%.

### 4.2. Studies of the Effect of Temperature on the TPC in P Comparing Two Heating Methods: Microwave (M) and Thermostatic Bath (TB)

In Table 5, it is possible to see that there is a decrease in the measured TPC values when the samples of P are subjected to heating both in a microwave synthesis platform and in a thermostatic bath. It is clear that TPC decay is present in all the TB tests and only in M at 80 °C. In contrast, there is no decay for TPC in M at 40 °C and M at 60 °C, providing a stable environment for our experiments.

Studies that have investigated the impact of temperature on polyphenol yield are mainly based on drying fruits and vegetables and extracting polyphenols from them. These studies have provided information on the thermal lability of plant polyphenols from different plant materials, which have indicated that external heat induces the loss of polyphenol content [41,42].

Thermal degradation is the most common mechanism explaining the drop in polyphenol yield during high-temperature extractions, as conventional extraction studies have shown that thermal degradation generally occurs at 80 °C [43]. However, it is important to consider that the duration of heating, storage conditions before heating, and exposure to oxygen play important roles in determining the effect of temperature on polyphenols [44].

It is also important to note that antioxidants can not only oxidize in the face of temperature increases; they can also degrade. In research carried out by Palma et al., the results of the extraction of polyphenols with boiling methanol (65 °C) showed that catechin experienced a 40% reduction in its concentration [45], while Loncaric and collaborators demonstrated that increasing the temperature to 100 °C causes shorter half-lives of catechin and epicatechin [46].

It should be noted that the studies are not carried out directly on a specific type of antioxidant as has been carried out in this study, and they involve all the components of the fruits and/or vegetables studied. In this sense, it has been shown that extracts with a higher carbohydrate content and lower tannin purity tend to have decreased thermal stability [47]. Therefore, the effect of heating under the proposed conditions is carried out directly on the antioxidants, without room for other types of reactions and, consequently, other degradation products.

To understand the behavior of polyphenols during exposure to high temperatures, it is necessary to understand all the factors that affect this parameter. Thermal degradation of different types of polyphenols occurs at different temperatures but depends on the pretreatment, type of solvent, pH, treatment time, environment, and source of the material. Regarding the antioxidant capacity, it is possible to observe that it follows the same thermal degradation pattern as phenolic compounds [48].

In this study, it is observed that there is a decline in the TPC; however, when comparing these values with what is described in the literature, it is possible to see that the TPC value continues to be within the averages for antioxidant extracts. The lowest TPC value recorded in this experiment was for TB 80 °C at 60 min and corresponds to 87.5 ± 2.1 (Table 5) (EAGmg/g), while Chardonnay, Black seeds, and Crimson grape samples have a CFT of 200, 94, and 53 EAGmg/g in dry weight, respectively [34].

Regarding the ANOVA analysis of the results, three effects are significant (Table 6) since they have a *p*-value < 0.05, indicating that they are significantly different from zero with a confidence level of 95.0%. Furthermore, the model fit describes that the experimental design factors predict 70% of the TPC.

The literature consistently shows that increasing temperature and time significantly affects TPC. However, this study also shows that the method by which these compounds are heated has a significant effect.

Microwaves are electromagnetic fields ranging from 300 MHz to 300 GHz with two perpendicular oscillating fields: electric and magnetic [49]. It is considered a non-thermal and “green” technology since it conforms to the criteria established by the Environmental Protection Agency (EPA) due to reduction in reaction times, energy consumption, use of solvents, and CO2 emissions in comparison with conventional heating methods [43].

Therefore, microwave equipment is a non-contact heat source that can provide more effective heating, accelerating energy transfer and reducing the thermal gradient [50]. Grigoras et al. (2012) [51] conducted a comparative study of conventional methods, maceration, and extraction using pressurized liquid, ultrasound, and microwaves. The microwave-assisted methodology provided the highest concentration of bioactive compounds in the apple extract. Boukroufa et al. (2015) [52] presented a study from laboratory to pilot scale for the extraction and separation of volatile and non-volatile compounds from boldo leaves using microwaves, concluding that this provided lower energy and time consumption because hydrodistillation required 30 min to initiate azeotropic distillation, while microwave-assisted extraction only required 5 min. This research indicates that microwaves quickly raise the volumetric temperature without overheating the surface, provide better process control, and achieve greater energy efficiency than conventional heating methods [53]. Some authors suggest that electric fields can alter the conformation of molecules, which are susceptible to structural changes that affect their functionality [54,55], as microwaves do not possess the energy necessary to break covalent bonds [35,56].

When comparing traditional and novel thermal processes, the discussion should focus on matching the temperature profiles since the results do not depend on any given mathematical model but on thermodynamic and molecular parameters [57]. Finally, it should be noted that microwave heating is an isothermal process where the temperature of the samples is uniform. In addition, the heating occurs due to the stretching and contraction of the bonds of the molecules with a high dielectric constant (water). In contrast, in conventional heating, the thermal gradient causes a higher temperature on the surface of the reactor and, therefore, the stretching of the bonds of all the molecules (in this case, the phenolic groups), giving them energy to activate oxidation reactions [58].

In Figure 2a, for the M 40 °C and M 60 °C runs, there is no significant degradation of the TPC, which is why the data do not fit any reaction model and have a dispersion within the standard deviation.

The linear regression plots (Table 7) were fitted with a higher coefficient of determination to the equation TPC = k t + TPC0 in which TPC corresponds to the molar concentration of equivalent polyphenols of gallic acid, therefore following a zero-order reaction model for TB 40 °C and TB 60 °C; however, a change is seen in the degradation mechanism for TB 80 °C, adjusting to a first order linear regression, which indicates that at this temperature the concentration of one species is affecting the degradation mechanism oxidation. This same behavior is observed for M80 °C, which fits a first-order linear regression.

It has been shown that flavonoids’ antioxidant activity is due to aromatic OH groups (phenols). Polyphenols’ chemical properties, in terms of the availability of phenolic hydrogens as hydrogen donor radical scavengers, suggest that they will have antioxidant activity [59].

Although the degradation rate depends on temperature, the number of soluble solids, and pH, several authors report that these kinetics follow first-order kinetics [60,61,62]. Zapata and collaborators (2001) showed that the thermal degradation of phenolic compounds follows first-order kinetics in the range of typical food processes (70–90 °C). The antioxidant activity of the extracts is directly related, following the same pattern [48], and Loncaric and collaborators (2018) demonstrate that the thermal degradation of catechin and epicatechin follows first-order kinetics [46].

The reducing capacity of flavan-3-ol involves a complex oxidation process that varies depending on the pH; it is produced by a consecutive proton donation mechanism by which phenol is converted into a phenoxyl radical (semiquinone), which is reversible, followed by electron transfer by which this radical is converted into quinone, which is irreversible [63]. These structures are almost identical to the original flavan-3-ol and cannot be detected by conventional methods such as thiolysis [41].

It is inferred that in TB at 40 °C and 60 °C, the oxidation mechanism occurs by deprotonating the benzene catechol ring and following zero-order kinetics. Therefore, it is inferred that this oxidation occurs without depending on the concentration of the species.

For TB80 °C and M80 °C, the oxidation mechanism follows two steps: deprotonation and electron transfer. These results agree with previous research in which it is shown that thermal degradation in general for polyphenols occurs at 80 °C [43]. In comparison, the degradation of condensed tannins remains low at temperatures that do not exceed 50 °C [41]. Let us consider that water contains approximately 9 mg/L of oxygen in a saturated state. Ethanol can contain up to 40 mg/L (under standard pressure and temperature conditions) [64]; it is important to highlight that the reactors are closed in this study. The reaction occurs under reflux, which indicates that there may be a redissolution of oxygen. Low temperatures have been considered to inhibit the degradation of active ingredients. However, low temperatures significantly increase operation times [41]. Therefore, this study seeks to use the highest possible temperature with a non-significant loss of TPC in microwaves, which is 60 °C, demonstrating that this method is optimal for heating polyphenols in contrast to the thermostatic bath.

### 4.3. Feasibility of Depolymerization of P Whit GA, Reaction Optimization and Kinetics Analysis

Table 8 describes the experimental design and results obtained for optimizing the P depolymerization reaction. In general, the reaction is feasible for all experimental runs, with an RY over 70% and, under optimized conditions, around 100%.

In the case of RY, there are four significant effects: the percentage of ethanol (S); the P/S ratio; and, in turn, the interaction between them; and finally, the interaction between P/S and P/GA (Table 9).

The chemical methods investigated for the depolymerization of polymeric CT with the addition of (+)-catechin, (−)-epicatechin, and (−)-epigallocatechin gallate as a chain-disrupting nucleophile pose a significant limitation in their use due to their high costs. In addition, they include the use of catalysts or alcoholic solutions of mineral acids that raise problems in terms of their toxicity, flammability and environmental impact [23,65]. Due to this, in this work, it is proposed to use a highly accessible and low-cost nucleophile, such as GA, and a GRAS solvent such as ethanol.

However, chemical reactivity can change dramatically in different solvents, and it is not always clear which solvent properties will affect reaction rates [66]. In solution, all participants in a chemical reaction are solvated, both reactants and products, including intermediates and transition states. However, the system under study does not represent a solution but rather a polymeric dispersion, in which the dispersed particles have molecular sizes of the order of microns, while a molecule of water or ethanol represents less than 1 nm. However, as described in the literature, CT is soluble in water and ethanol; this is due to the large number of phenols, which can form hydrogen bonds with this type of polar protic solvents, which does not happen with their monomers, which have a very low solubility in water [67].

Then, the ethanol/water ratio is influential in three respects: in the solubility of the reactants, where, on the one hand, the GA dissolves in both solvents; in the solubility of the products, where the catechin and epicatechin monomers are better solubilized as the proportion of ethanol increases; and finally, in the dielectric constant (ε), which increases as the water content increases. The ε represents the ability of the solvent to dissociate a solute into ions through interatomic and intermolecular interactions [68]. This last variable becomes very important in this context in which microwaves assist the reaction. Some researchers have reported that the degradation of CT under microwaves occurs at a temperature above 60 °C, and the mDP of the products decreases gradually [27,69].

Water, methanol, and ethanol have ε of 79.99, 33.30, and 24.55 (in standard pressure and temperature conditions), respectively [70], and theoretically, 80% ethanol in 20% water has an ε of 35.64, similar to methanol—that is, the solvent generally used in the depolymerization of condensed tannins [21].

So, although, in a microwave-assisted reaction, the greater the ε of the solvent, the greater the reaction rate is expected to be; on the other hand, we have the opposite effect on the solvation of the products. This premise can be inferred based on the MPD results. For the MPD, it is observed that only the percentage of ethanol has a significant effect, this is because the solvent has an effect on the solubility of the products, which is reflected in their agglomeration; so, the more aggregated the products are, it means that their affinity with the solvent decreases; therefore, they will look for a way to reduce their energy again by condensing and reversing the reaction, impacting a decrease in the RY. Stepwise polymer growth occurs slowly in condensation reactions, but eventually, long chain polymers are formed [71].

In water, as the reaction proceeds, the particle size decreases, so their solvation should increase; however, the opposite effect occurs since, although CT is soluble in water, the monomers do not have aqueous solubilities at acidic pH (1.7) GA 86.76 mg/mL catechin and epicatechin 5.59 mg/mL [72]; this effect can be seen by increasing the percentage of ethanol from 0 to 50% *v*/*v*, in which the MDP decreases drastically. Although most reactions occur in aqueous media, much remains to be understood regarding their interactions, especially with other more complex particles, considering their sensitivity to factors such as temperature or the presence of an electric field [73]. This last factor becomes important in this reaction since this external “stress” factor is associated with ionic migration processes, the change in the orientation of the molecular dipole moments, the length of bonds in a molecule, and the distribution of charges in an atom. More information about how depolymerization occurs can be obtained by analyzing the optimized reaction over time.

The reaction’s efficiency is approximately 100%, likely due to the suitable nucleophile and the electromagnetic wave inducing a polarization reaction in the macromolecular structure. The polar bonds quickly absorbed the microwave energy, leading to a decrease in activation energy, an increase in reaction activity within the polar bonds, and an enhancement of the depolymerization effect [27,69].

These results are mainly because depolymerization occurs by the formation of a flavan-3-ol carbocation followed by the formation of an adduct with the GA nucleophile, which forms an ester between catechin/epicatechin, a GA homologous to catechin/epicatechin gallate. However, under the reaction conditions, this ester is hydrolyzed, giving rise to epicatechin monomer and the recovery of GA [74].

Therefore, the formation of epicatechin does not depend on the concentration of AG or P in this case, following a constant rate formation with zero-order kinetics (Figure 3c) since it relies on the formation of the carbocation, the nucleophilic attack and the hydrolysis of the ester consecutively, processes that are spontaneous in these reaction conditions. However, it is complex to understand all the processes that may be involved in the formation of the final products; it has been described in the literature that the main reaction products can follow several fragmentation routes, including quinone cleavage, retro-cleavage, Diels–Alder, heterocyclic ring fission, and gallate loss [24,40].

On the other hand, it is possible to observe an abrupt drop in the concentration of PR during the first 5 min of the reaction, which results in a linear adjustment of second-order kinetics. The same adjustment is observed for GA, which could be consumed by forming adducts with cyanidins formed by the oxidation of catechin and epicatechins [24,40,65].

When analyzing the reaction products by UHPLC in the absorption range of 545 nm, seven chromatographic peaks at 36.8 can be seen: 39.6; 41.6; 43.6; 44.2; 45.0; and 49.1 min (Figure S1). These peaks do not appear in the suspension at 0 time and increase over time with zero-order kinetics (Table S1). This absorption range is typical for cyanidings, one of the main formation products of the cleavage of procyanidins in acid alcohol [40].

Ethanol recovery is crucial for enhancing the sustainability and cost-effectiveness of our process. This solvent can be efficiently recovered via vacuum evaporation using a rotary evaporator, a well-established technique for solvent recovery in polyphenol extraction [75,76]. The solvent can be further purified through nitrogen bubbling and ultrasonication, which ensures minimal solvent loss and prevents oxidation of bioactive compounds [77]. Studies indicate that over 90% of ethanol can be recovered under optimized vacuum conditions, significantly reducing solvent consumption [75,78].

Implementing solvent recovery systems reduces operational costs and minimizes environmental impact, aligning with sustainability goals in green chemistry applications [79,80].

### 4.4. Optimized Depolymerization Color Analysis

It is possible to observe a variation in the suspension’s color as a function of the reaction time, which changes from orange-brown, the color of P, to an intense dark red (Figure 4a); this is reflected in the change of the ∆E* and ∆C* parameters (Figure 4d and Figure 4e, respectively).

The results obtained from the color analysis support the hypothesis of cyanidin formation due to the appearance of its typical red color in acidic pH [81]. This is reflected in the increase in the a* parameter (Figure 4c). As the reaction time progresses, a decrease in the L* parameter is observed (Figure 4b) due to the increase in cyanidin concentration [82]. All parameters follow zero-order kinetics (Table 11), consistent with the kinetics of cyanidin formation (Table S1), so the color in this reaction can be considered a parameter of the reaction’s efficiency in terms of cyanidin formation as they have a constant formation rate independent of the concentration of other molecules [83].

### 4.5. Discussion of Antioxidant Capacity

It is possible to observe an increase in the TPC for P-Dep compared to P (Table 12), which is highly beneficial for this study. The P-Dep inhibited the DPPH● radical by 88.2%, higher than that reported by Li and collaborators (2023), where the maximum DPPH● elimination rate in their experimental design was 81.26% [27]. Furthermore, it is also possible to see that the inhibition percentage for ABTS●+ radicals is higher for P-Dep by 4.2%, exceeding 90% inhibition.

This phenomenon is due to the “break” of the interflavanic bonds, which makes it possible to expose antioxidant groups (hydroxyls) and promote the release of more electrons. Added to this effect, the decrease in molecular size promotes more significant binding to DPPH● sites, avoiding the steric impediments that CT has [27,84].

Previous studies on depolymerization have mainly focused on optimizing experimental conditions and separating and identifying the resulting compounds. As a result, these studies often overlook assessing the antioxidant capacity of the obtained products [20,21,23,24,25]. However, research by Wen et al. has shown an increase in antioxidant capacity following depolymerization. The free radical scavenging ability of the antioxidant was quantified using the IC50 index, which represents the concentration needed to eliminate 50% of DPPH● radicals. The IC50 values for proanthocyanidins decreased as the degree of polymerization increased, ranging from 50.6 to 54.3 mg/mL [22].

### 4.6. Economic Analysis and Future Perspectives

To evaluate the feasibility of the microwave-assisted depolymerization process of grape seed condensed tannins using gallic acid as a nucleophile, we assessed the market value of the products and the cost of consumables and reagents.

The primary product of the depolymerization process is epicatechin, a flavanol with significant antioxidant properties used in pharmaceuticals, nutraceuticals, and cosmetics. The market price of high-purity epicatechin ranges between USD 200 to USD500 per gram, depending on the supplier and purity grade [79,85].

The major consumables in our process include ethanol (solvent) and gallic acid (nucleophile). Industrial-grade ethanol costs approximately USD 1 to USD 3 per liter, while gallic acid is priced at around USD 50 to USD 100 per kilogram [80,86]. Based on our optimized reaction conditions (80% *v*/*v* ethanol, 10 mg/mL polymeric tannins, and 5.88 mg/mL gallic acid), the cost per liter of reaction mixture is relatively low compared to the commercial value of epicatechin.

Transitioning from laboratory-scale experiments to pilot-scale production requires designing microwave reactors capable of handling larger volumes while maintaining reaction uniformity [76,78,79]. Developing a continuous flow microwave-assisted extraction system could improve production rates and process efficiency. Continuous microwave processing has been successfully applied in polyphenol extraction and enhances reaction reproducibility [76,79,87]. After depolymerization, purification steps such as membrane filtration and chromatography will be employed to isolate high-purity epicatechin. Advanced spectroscopic and chromatographic techniques will confirm the structural integrity and antioxidant activity of the final product [79,80]. Given the food-grade nature of our approach, regulatory approval pathways must be explored. This will facilitate the incorporation of epicatechin into food, nutraceutical, and pharmaceutical formulations [79,85].

## 5. Conclusions

This study demonstrates that grape seed condensed tannins can be efficiently depolymerized under food-grade conditions using microwave-assisted heating, ethanol as a Generally Recognized as Safe (GRAS) solvent, and gallic acid as a nucleophile. Thermal analysis confirms that total polyphenol content (TPC) degradation follows first-order kinetics above 80 °C in microwaves, while depolymerization follows second-order kinetics, yielding epicatechin as the primary product with zero-order formation kinetics. Optimization of the reaction conditions—80% *v*/*v* ethanol, 10 mg/mL polymeric tannins, and 5.88 mg/mL gallic acid—resulted in a 99.9% reaction yield, with a mean particle diameter of 5.7 nm. The final product retained a high TPC (297.3 ± 15.9 mg EAG/g) and strong antioxidant activity, inhibiting ABTS●+ and DPPH● radicals by 93.5 ± 0.9% and 88.2 ± 1.5%, respectively. These findings provide a sustainable and scalable approach for valorizing food waste by converting condensed tannins into bioavailable antioxidant compounds, reinforcing the potential of microwave-assisted methodologies in food chemistry applications. The main reaction product of this study is the epicatechin, a high-value compound, while maintaining low process costs. Solvent recovery through vacuum evaporation achieves over 90% efficiency, further enhancing sustainability. Future developments focus on scaling up with continuous flow systems, improving purification methods, and ensuring regulatory compliance to support industrial applications in food, nutraceuticals, and pharmaceuticals.

## Figures and Tables

**Table 1:** Experimental design for studies of thermal resistance and microwave behavior of grape seed condensed tannins (CT).

	Definition and Units	Levels
FACTORS	Temperature (°C)	40; 60; 80
	Time (min)	10; 30; 60
	Heating method	M; TB
RESPONSE	TPC (mgEGA/g)	-

**Table 2:** Experimental design for optimization of the chemical depolymerization reaction.

	Definition and Units	Levels
FACTORS	Percentage of ethanol (S) (% *v*/*v*)	0; 20; 50; 80; 100
	Polymers/Solvent ratio (P/S) (% *m*/*v*)	0.1; 1
	Polymers/Nucleophile ratio (P/GA) (mol/mol)	0.5; 1
RESPONSE	Reaction Yield (RY) (%)	-
	MPD (nm)	-

**Table 3:** Physicochemical characterization of initial grape seed condensed tannin (P).

Parameter	Value
TPC (EAGmg/g)	234.62 ± 0.17
MPD (µm)	16.55 ± 1.47
PDI	3.50 ± 1.59
Trolox equivalent (mmol/L)	8.8 ± 0.3
ABTS inhibition (%)	89.30 ± 2.27
DPPH inhibition (%)	87.59 ± 1.12

**Table 4:** Molar concentration and percentage of the main component chemical structures of initial condensed grape seed tannins (P) and their resulting species in the cysteamine depolymerization procedure (P-Tiol), *n* = 3.

Chemical Structure	Retention Time	Concentration (mol/L)		Amount (%)	
		P	P-Tiol	P	P-Tiol
Gallic Acid	5.0	1.16 × 10^−5^ ± 1.76 × 10^−7^	1.73 × 10^−5^ ± 5.07 × 10^−6^	1.03 ± 0.20	1.00 ± 0.33
Catechin	15.4	4.94 × 10^−5^ ± 5.51 × 10^−7^	1.34 × 10^−4^ ± 4.03 × 10^−6^	4.41 ± 0.06	7.74 ± 0.02
Catechin-tiol	18.2	0	4.76 × 10^−5^ ± 4.46 × 10^−6^	0	2.75 ± 0.45
Epicatechin	27.5	2.98 × 10^−5^ ± 3.51 × 10^−7^	1.04 × 10^−4^ ± 1.33 × 10^−6^	2.66 ± 0.03	6.02 ± 0.28
Epicatechin-tiol	28.4	0	9.89 × 10^−4^ ± 6.49 × 10^−5^	0	57.19 ± 5.62
Polymers	35 to 45	1.03 × 10^−3^ ± 9.59 × 10^−6^	4.38 × 10^−4^ ± 9.72 × 10^−5^	91.90 ± 0.25 ^1^	25.31 ± 6.63 ^2^
Terminal units	-	-	1.59 × 10^−4^	-	-
Extension units	-	-	1.04 × 10^−3^	-	-
RY					57.50
mDP					7.53

^1^ Amount of polymers added to the reactor. ^2^ Amount of polymers remained after reaction.

**Table 5:** Multilevel 3^2^ × 2^1^ factorial experimental design for heating P on TPC over time at different temperatures through two heating methods. Ethanol solvent 50% pH 3, *n* = 3.

Sample	Heating Method	Temperature (°C)	Time (min)	TPC (GAEmg/g)
2	M	40	10	228.8 ± 4.2
8	M	40	30	221.0 ± 2.3
13	M	40	60	226.4 ± 1.9
17	M	60	10	224.0 ± 2.0
15	M	60	30	217.8 ± 11.7
12	M	60	60	219.9 ± 8.4
18	M	80	10	194.4 ± 4.9
4	M	80	30	163.6 ± 14.8
7	M	80	60	111.0 ± 5.2
9	TB	40	10	223.4 ± 1.5
16	TB	40	30	199.4 ± 1.5
1	TB	40	60	139.4 ± 0.9
5	TB	60	10	220.8 ± 1.1
14	TB	60	30	199.4 ± 1.5
11	TB	60	60	109.7 ± 2.9
6	TB	80	10	162.6 ± 1.3
10	TB	80	30	142.0 ± 1.2
3	TB	80	60	87.5 ± 2.1

**Table 6:** Analysis of variance for TPC—Type III sum of squares. This analysis considers the effect of the variables separately as well as their interactions.

Sources (Factors)	F-Ratio	*p*-Value
Temperature	29.66	0.0000 *
Time	26.82	0.0000 *
Heating method	5.78	0.0209 *
Temperature–time interaction	1.63	0.1864
Temperature–heating method interaction	1.72	0.1923
Time–heating method interaction	2.12	0.1335
R^2^	69.9%	
Adjusted R^2^	64.5%	

* *p* < 0.05.

**Table 7:** Mathematical adjustment of the results obtained from TPC as a function of time, temperature, and heating method (Table 6).

Samples Set	Adjusted Model	Equation	R^2^
TB 40 °C	Order 0	TPC = −9.81t ^1^ + 1.45	0.9072
TB 60 °C	Order 0	TPC = −1.27t + 1.46	0.9079
TB 80 °C	Order 1	Ln(TPC/TPC0 ^2^) = −0.0137 t – 0.1259	0.9477
M 40 °C	-	-	-
M 60 °C	-	-	-
M 80 °C	Order 1	Ln(TPC/TPC0) = −0.0113 t – 0.1339	0.9897

^1^ Time. ^2^ TPC at time 0.

**Table 8:** Experimental design and results obtained for the optimization of the P depolymerization reaction.

Sample	S (% *v*/*v*)	P/S Ratio (% *m*/*v*)	P (mol/L)	P/GA Ratio (mol/mol)	GA (mol/L)	RY (%)	MPD (nm)
15	100	1.0	3.4 × 10^−2^	1.0	3.4 × 10^−2^	99.5	6.5
5	80	1.0	3.4 × 10^−2^	1.0	3.4 × 10^−2^	99.9	5.7
19	50	1.0	3.4 × 10^−2^	1.0	3.4 × 10^−2^	99.6	5.5
11	20	1.0	3.4 × 10^−2^	1.0	3.4 × 10^−2^	87.4	2524.1
16	0	1.0	3.4 × 10^−2^	1.0	3.4 × 10^−2^	69.6	8399.0
8	100	1.0	3.4 × 10^−2^	0.5	1.7 × 10^−2^	88.9	933.1
17	80	1.0	3.4 × 10^−2^	0.5	1.7 × 10^−2^	89.6	643.2
3	50	1.0	3.4 × 10^−2^	0.5	1.7 × 10^−2^	90.8	75.9
9	20	1.0	3.4 × 10^−2^	0.5	1.7 × 10^−2^	79.6	3608.0
1	0	1.0	3.4 × 10^−2^	0.5	1.7 × 10^−2^	71.9	6171.7
13	100	0.1	3.4 × 10^−3^	1.0	1.7 × 10^−2^	87.6	1582.7
14	80	0.1	3.4 × 10^−3^	1.0	3.4 × 10^−3^	87.8	1104.6
20	50	0.1	3.4 × 10^−3^	1.0	3.4 × 10^−3^	89.7	182.0
18	20	0.1	3.4 × 10^−3^	1.0	3.4 × 10^−3^	98.9	376.7
2	0	0.1	3.4 × 10^−3^	1.0	3.4 × 10^−3^	98.2	1755.2
4	100	0.1	3.4 × 10^−3^	0.5	1.7 × 10^−3^	98.7	708.8
7	80	0.1	3.4 × 10^−3^	0.5	1.7 × 10^−3^	99.0	550.7
12	50	0.1	3.4 × 10^−3^	0.5	1.7 × 10^−3^	99.0	86.5
10	20	0.1	3.4 × 10^−3^	0.5	1.7 × 10^−3^	98.7	1637.1
6	0	0.1	3.4 × 10^−3^	0.5	1.7 × 10^−3^	97.3	5951.6

**Table 9:** Analysis of variance for reaction yield and mean particle size—Type III sum of squares.

RY			MPD	
Sources (Factors)	F-Ratio	*p*-Value	F-Ratio	*p*-Value
A: Ethanol (% *v*/*v*)	8.39	0.0134 *	16.35	0.0016 *
B: Polymers/Solvent ratio (% *m*/*v*)	13.50	0.0032 *	1.21	0.2938
C: Polymers/Gallic acid ratio (mol/mol)	0.05	0.8287	0.33	0.5754
A–B Interaction	23.42	0.0004 *	3.94	0.0706
A–C interaction	0.06	0.8173	0.31	0.5853
B–C interaction	9.55	0.0094 *	0.20	0.6621
A–B–C interactions	4.49	0.0557	2.69	0.1270
R^2^	83%		68%	
Adjusted R^2^	73%		49%	

* *p* < 0.05.

**Table 10:** Equations resulting from the linear regressions for the concentration of the main species during depolymerization in the mixed depolymerization product suspension (P-Dep).

Molecule	Adjusted Model	Equation	R^2^
GA	Order 2	(1/C ^1^ − 1/C0 ^2^) = 0.108t	0.9638
Ep	Order 0	C = 2.0 × 10^−4^t	0.9622
P	Order 2	(1/C − 1/C0) = 1.98 × 10^−2^t	0.8915

^1^ Concentration of the molecule over time. ^2^ Concentration initial of the molecule.

**Table 11:** Equations resulting from the linear regressions for the color parameters during depolymerization.

Parameter	Adjusted Model	Equation	R^2^
L*	Order 0	L* = −6.29 × 10^−1^t + 8.48 × 10^1^	0.9294
a*	Order 0	a* = 5.05 × 10^−1^t + 1.14	0.9329
∆E*	Order 0	∆E* = 7.91 × 10^−1^t + 1.13	0.9295
∆C*	Order 0	∆C* = 4.80 × 10^−1^t − 3.17 × 10^−2^	0.9046

**Table 12:** Total phenolic content and antioxidant capacity of P, P-Dep, and AG suspensions according to ABTS●+ and DPPH● assay.

Sample	% ABTS●+ Inhibition	% DPPH● Inhibition	TPC (mgEGA/g)	Eq. Trolox (mmol/L)
P	89.3 ± 2.3	87.6 ± 1.1	234.6 ± 0.2	8.8 ± 0.3
P-Dep	93.5 ± 0.9	88.2 ± 1.5	297.3 ± 15.9	9.2 ± 0.7
AG	94.9 ± 1.1	86.7 ± 1.3	108.8 ± 7.1	9.4 ± 0.6

## Data Availability

The original contributions presented in this study are included in the article/Supplementary Material. Further inquiries can be directed to the corresponding author.

## Supplementary Materials

The following supporting information can be downloaded at https://www.mdpi.com/article/10.3390/polym17050682/s1, Figure S1: UHPLC-VIS chromatogram at 545 nm for TSD samples after 60 minutes of reaction. Table S1: Linear regressions for the concentration of cyanidin species absorbing at 545 nm, during depolymerization.

## Abbreviations

CT: condensed tannins; P: polymeric condensed tannins; P-Dep: Depolymerized condensed tannins; P_A_: polymeric condensed tannins added to reactor; P_R_: polymeric condensed tannins remaining after depolymerization; P-Tiol: polymeric condensed tannins after thiolysis; Ep: epicatechin; Ca: catechin; GA: gallic acid; TPC: total polyphenols content; SN1: unimolecular nucleophilic substitution reaction; Nü: nucleophile; mDP: its main degree of polymerization; M: microwave reaction platform; TB: thermostatic bath; S: solvent reaction; MPD: Mean particle diameters; PDI: polydispersity index; RY: reaction yield.
