# Supplementary material for: Food-Grade Microwave-Assisted Depolymerization of Grape Seed Condensed Tannins: Optimizing the Reaction Using Gallic Acid as a Nucleophile
Source: Polymers (Basel). 2025 Mar 4;17(5):682. doi: 10.3390/polym17050682 (PMC11902613; doi:10.3390/polym17050682)
Supplement: Supplementary file 1 [file polymers-17-00682-s001.zip › Table S1.pdf]

Table S1. Linear regressions for the concentration of cyanidin species absorbing at 545 nm, during depolymerization.

Molecule retention time	Adjusted model	equation	R^2
36.8	Order 0	$A^1 = 0.0129t^2 - 0.0305$	0.9871
39.6	Order 0	$A = 0.0479t - 0.1748$	0.9738
41.6	Order 0	$A = 0.0111t - 0.0469$	0.9186
43.6	Order 0	$A = 0.0014t - 0.0075$	0.8754
44.2	Order 0	$A = 0.0022t - 0.0046$	0.9263
45.0	Order 0	$A = 0.0056t - 0.0244$	0.9432
49.1	Order 0	$A = 0.1935t - 0.6811$	0.9706

[1] Area at retention time, [2] time reaction.
