# Supplementary figures and images for: Food-Grade Microwave-Assisted Depolymerization of Grape Seed Condensed Tannins: Optimizing the Reaction Using Gallic Acid as a Nucleophile
Source: Polymers (Basel). 2025 Mar 4;17(5):682. doi: 10.3390/polym17050682 (PMC11902613; doi:10.3390/polym17050682)

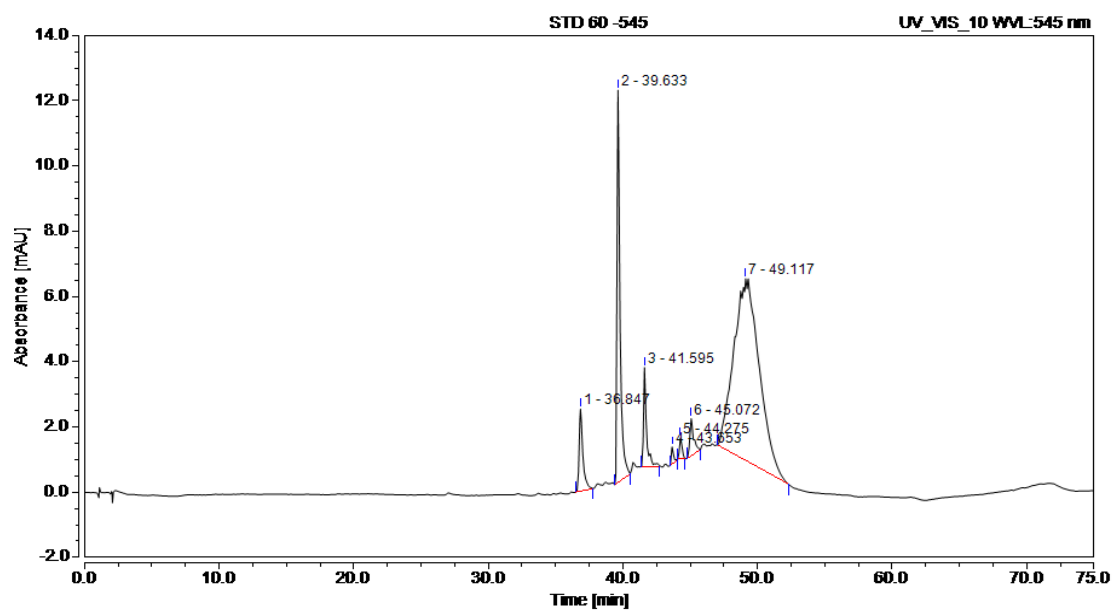

Figure S1. UHPLC-VIS chromatogram at 545 nm for TSD samples after 60 minutes of reaction.

Supplement: Supplementary file 1 [file polymers-17-00682-s001.zip › Figure S1.pdf]
